# Nonhuman primates’ tissue banks: resources for all model organism research

**DOI:** 10.1007/s00335-021-09925-w

**Published:** 2021-10-26

**Authors:** Claire Witham, Sara Wells

**Affiliations:** Medical Research Council, Centre for Macaques, Porton Down, Salisbury, Wiltshire, SP40JQ UK

## Abstract

Biobanks containing tissue and other biological samples from many model organisms provide easy and faster access to ex vivo resources for a wide-range of research programmes. For all laboratory animals, collecting and preserving tissue at post-mortem is an effective way of maximising the benefits of individual animals and potentially reducing the numbers required for experimentation in the future. For primate tissues, biobanks represent the scarcest of these resources but quite possibly those most valuable for preclinical and translation studies.

## Introduction

Archives of model organism tissues have been vital for a multitude of studies including those elucidating biological mechanisms (Adissu et al. 2014) and now provide the potential for much wider, complex, comparative translational studies (Brubaker and Lauffenburger 2020). However, establishing and curating tissue collection is a significant undertaking. Careful evaluation of the long-term resources needed to support archives as well as the investment in the set-up and ongoing sample collection need to be balanced against how irreplaceable the samples are and the benefits in terms of reducing animal numbers. The benefits greatly outweigh the cost for repositories of many specific disease tissues (https://searchbreast.org), tissue from large fundamental biology projects (www.mousephenotype.org) and samples from the least common, most sensitive laboratory animal species such as non-human primates.

## Primate biobanks

The most common non-human primate laboratory species are rhesus and cynomolgus macaques and marmosets. Unlike other biobanks from animals such as the mouse, primate biobanks represent more diverse and non-standardised samples whose availability often changes as unique samples are used. The age, sex and life-experience of individual animals contributing to these archives are determined by their primary scientific purpose, whether experimental or breeding. Sample collection is likely to be sporadic, over many years, performed by different individuals and only include the addition of samples representing a small number of animals at any one time. To reduce variation, standard operating procedures (SOPs) for tissue collection are used to ensure consistent sampling techniques as well as the cataloguing of full metadata including age, sex and health records.

Without doubt, the samples contained within primate biobanks constitute invaluable resources for translational research, especially where human samples are scarce or for the timely extrapolation of data from other model organisms to the order of primates. However, it should be noted that most laboratory primates are not kept until geriatric ages and their living environment, including protection from disease and availability of a standard and consistent diet, may not always mimic the human condition.

For those wishing to access these valuable resources, Table 1 summarises the offerings from 4 major international primate biobanks.

**Table 1:** Primate biobanks accessible for biomedical researchers

	Biomedical primate research centre, BPRC, The Netherlands	National institute of aging, NIH, USA	Deutsches primatenzentrum: DPZ, Germany	Medical research council, centre for macaques, UK
Species	Various	Various	Various	Rhesus macaques
Frozen tissue (− 80)	x	x	Upon enquiry	x
Fixed blocks	o	x	Upon enquiry	x
Slides	o	x	Upon enquiry	x
DNA	x	x	x	x
Link	www.bprc.nl/en/biobank	www.nia.nih.gov/research/dab/nonhuman-primate-tissue-bank-handbook	www.dpz.eu/en/unit/primate-genetics/about-us/service/gene-bank-of-primate	www.har.mrc.ac.uk/harwell-news/mrc-centre-for-macaques-biobank/
Comment	Europe’s largest biobank	Restricted to USA only		

## Ethical review and governance

Although the samples within primate tissue banks often represent the opportunistic collection of tissues following an experiment or at the end of the breeding life-span of an animal, their use still required careful ethical governance. It is recommended good practice for research institutes to review the sources of animal-derived biologicals even if the animal samples have been collected many years ago (Berdoy et al. 2015). This review should include information gathered on the welfare standards of the contributing facilities as well as a rigorous scientifically justification for the experiments planned. The samples from genetically diverse NHP colonies are irreplaceable and it is the duty of all users to ensure every experiment delivers valuable results which could not be obtained by any non-animal alternative.

## Zoonotic diseases and health and safety

Unlike the specific pathogen-free environments that mice are commonly bred in, NHP colonies have the potential to carry a number of zoonotic diseases. Primate biobanks will provide details of health screens performed on the animals near to or at the time of post-mortem; however, there is the potential that primates can carry a number of pathogens which are transmissible to humans. These include Macacine herpesvirus 1 (herpes B virus) and more recently COVID-19. It is strongly recommended that samples are handled using the appropriate biosafety containment similar to those used for human biobanks (Roux et al 2021).

## Summary

As the ability of the research community to model data and concepts from model organism research to human cellular and physiological systems, it is likely that the usefulness of primate biobanks will increase. This can only be sustained by the long-term investment in not only preserving samples from these highly-valued and sensitive animals but also by the concomitant collection of health and behavioural data and metadata. This is a challenge as non-human primates may be kept for a number of years and experience vastly different housing and experiment conditions. However, the combination of tissue, health and phenotype data and a greater understanding of NHP genomics provide a rich and growing resource for comparative human and primate studies.
